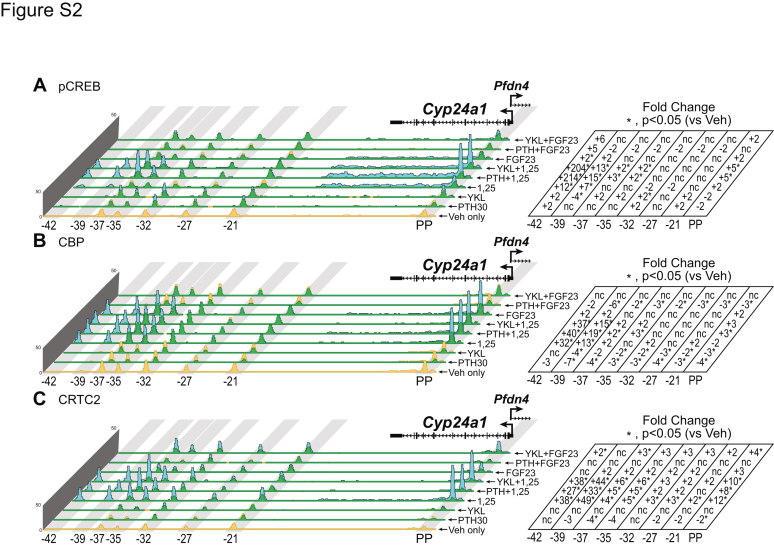# Rapid genomic changes by mineralotropic hormones and kinase SIK inhibition drive coordinated renal *Cyp27b1* and *Cyp24a1* expression *via* CREB modules

**DOI:** 10.1016/j.jbc.2022.102559

**Published:** 2022-09-30

**Authors:** Mark B. Meyer, Nancy A. Benkusky, Seong Min Lee, Sung-Hee Yoon, Michael Mannstadt, Marc N. Wein, J. Wesley Pike

**Affiliations:** 1 Department of Biochemistry, University of Wisconsin-Madison, Madison, Wisconsin, USA; 2 Endocrine Unit, Massachusetts General Hospital, Harvard Medical School, Boston, Massachusetts, USA

**Keywords:** vitamin D, gene regulation, parathyroid hormone, cytochrome P450, ChIP-Seq, 1,25(OH)_2_D_3_, *Cyp27b1*, *Cyp24a1*, FGF23, salt-inducible kinases, bw, body weight, Ca, calcium, CBP, CREB-binding protein, ChIP-Seq, chromatin immunoprecipitation sequencing, CP, *Cyp27b1* promoter, CRTC, CREB-regulated transcription coactivator, FC, fold change, FGF23, fibroblast growth factor 23, H3K9ac, histone acetylation at histone H3 lysine 9, H3K27ac, histone acetylation at histone H3 lysine 27, HPBCD, hydroxypropyl β-cyclodextrin, iFGF23, intact FGF23, 1,25(OH)_2_D_3_, 1,25-dihydroxyvitamin D_3_, P, phosphorus, pCREB, phosphorylated (p-133) CREB, PP, promoter–proximal, pol II, polymerase II, PTH, parathyroid hormone, qRT–PCR, quantitative RT–PCR, RXR, retinoid X receptor, SIK, salt-inducible kinase, TF, transcription factor, VDR, vitamin D receptor

## Abstract

Vitamin D metabolism centers on kidney regulation of *Cyp27b1* by mineralotropic hormones, including induction by parathyroid hormone (PTH), suppression by fibroblast growth factor 23 (FGF23) and 1,25-dihydroxyvitamin D_3_ (1,25(OH)_2_D_3_), and reciprocal regulations for *Cyp24a1*. This coordinated genomic regulation results in production of endocrine 1,25(OH)_2_D_3_, which, together with PTH and FGF23, controls mineral homeostasis. However, how these events are coordinated is unclear. Here, using *in vivo* chromatin immunoprecipitation sequencing in mouse kidney, we demonstrate that PTH activation rapidly induces increased recruitment of phosphorylated (p-133) CREB (pCREB) and its coactivators, CBP (CREB-binding protein) and CRTC2 (CREB-regulated transcription coactivator 2), to previously defined kidney-specific M1 and M21 enhancers near the *Cyp27b1* gene. At distal enhancers of the *Cyp24a1* gene, PTH suppression dismisses CBP with only minor changes in pCREB and CRTC2 occupancy, all of which correlate with decreased genomic activity and reduced transcripts. Treatment of mice with salt-inducible kinase inhibitors (YKL-05-099 and SK-124) yields rapid genomic recruitment of CRTC2 to *Cyp27b1*, limited interaction of CBP, and a transcriptional response for both *Cyp27b1* and *Cyp24a1* that mirrors the actions of PTH. Surprisingly, we find that 1,25(OH)_2_D_3_ suppression increases the occupancy of CRTC2 in the M1 enhancer, a novel observation for CRTC2 and 1,25(OH)_2_D_3_ action. Suppressive actions of 1,25(OH)_2_D_3_ and FGF23 at the *Cyp27b1* gene are associated with reduced CBP recruitment at these CREB-module enhancers that disrupts full PTH induction. Our findings show that CRTC2 contributes to transcription of both *Cyp27b1* and *Cyp24a1*, demonstrate salt-inducible kinase inhibition as a key modulator of vitamin D metabolism, and provide molecular insight into the coordinated mechanistic actions of PTH, FGF23, and 1,25(OH)_2_D_3_ in the kidney that regulate mineral homeostasis.

Parathyroid hormone (PTH), fibroblast growth factor 23 (FGF23), and 1,25-dihydroxyvitamin D_3_ (1,25(OH)_2_D_3_) comprise the three primary mineralotropic hormones that orchestrate the regulation of mineral homeostasis in higher organisms *via* the skeleton, kidney, and intestine (1, 2, 3). Each of these hormones functions selectively in specific tissues to regulate the expression of genes whose products control (1) the differentiation and/or maturation of unique cell types, (2) bone formation, mineralization, and resorption, (3) the uptake of calcium (Ca) and phosphorus (P) by the intestine, and (4) internalization and/or secretion of Ca and P in multiple cell types including bone and kidney, all actions that serve to maintain dynamically the circulating levels of extracellular Ca and P. Because of unique and often opposing interactions at genes, each hormone also contributes to the transcriptionally and/or post-transcriptional modulation of the other two, serving either to stimulate or to feedback repress their production as well. Thus, PTH induces the levels of 1,25(OH)_2_D_3_ and FGF23, 1,25(OH)_2_D_3_ induces FGF23 and suppresses the levels of PTH secretion, and 1,25(OH)_2_D_3_ biosynthesis and FGF23 suppresses PTH and 1,25(OH)_2_D_3_, thereby achieving an appropriately exquisite and sensitive integration of mineral regulation by three different hormones (4, 5, 6).

During normal physiologic conditions, the synthesis of bioactive endocrine 1,25(OH)_2_D_3_ occurs exclusively in the kidney, where it is produced from its precursor substrate 25(OH)D_3_ and then released into the circulation (7). 1,25(OH)_2_D_3_ is synthesized by the 25(OH)D_3_-1α-hydroxylase enzyme, which is encoded by the *Cyp27b1* gene, expressed in proximal tubules, its concentration further modified through degradation by the 24-hydroxlase enzyme, which is encoded by the *Cyp24a1* gene, both of which contribute to the dynamic physiological control of 1,25(OH)_2_D_3_ in the blood (8, 9). As stated earlier, the levels of these renal enzymes are modulated through transcriptional control of the expression of both *Cyp27b1* and *Cyp24a1* genes, regulation modulated in reciprocal fashion by PTH, FGF23, and 1,25(OH)_2_D_3_ (10, 11). Indeed, PTH induces *Cyp27b1* and suppresses *Cyp24a1* expression, whereas both FGF23 and 1,25(OH)_2_D_3_ suppress the expression of the former and induce the latter, which is summarized schematically in Figure 1*A*.

**Figure 1:**
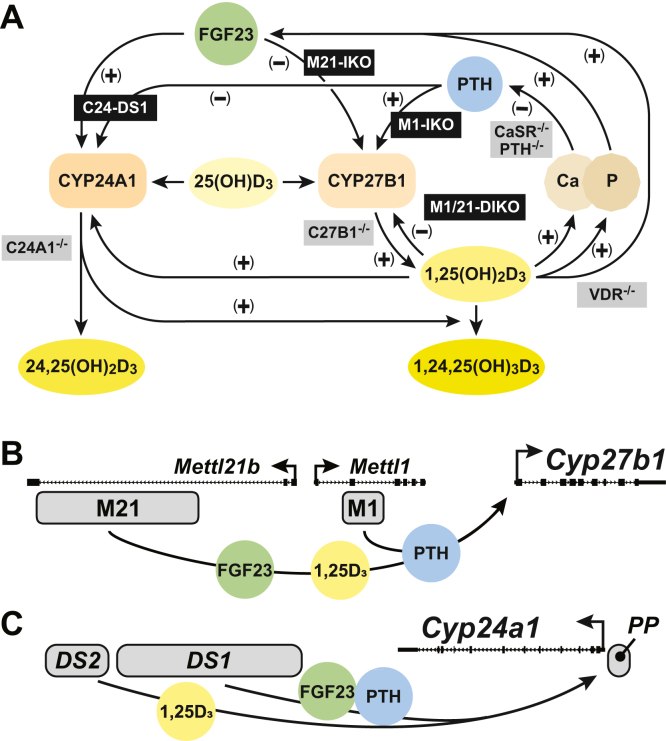
**Pathways and enhancers that control the expression of *Cyp27b1* and *Cyp24a1* in the kidney.***A*, schematic diagram for the regulation of vitamin D metabolism and serum calcium and phosphate homeostasis in the kidney. Pathways either increase (+) or decrease (−) in response to changing calcium and phosphate levels. Our genetic models (*black*) and previously existing models (*gray*) are overlaid on or near the pathways they disrupt. Schematic representation of PTH, FGF23, and 1,25(OH)_2_D_3_ actions on *Cyp27b1* (*B*) and *Cyp24a1* (*C*) through tissue-specific enhancers M1 and M21, which control *Cyp27b1* as well as DS1 (-21 kb to -32 kb) and DS2 (-35 kb to -37 kb) enhancers that control *Cyp24a1*. Figures adapted from recent studies (2, 15). DS, DownStream region of *Cyp24a1*; FGF23, fibroblast growth factor 23; 1,25(OH)_2_D_3,_ 1,25-dihydroxyvitamin D_3_; PTH, parathyroid hormone.

General observations that PTH was integral to the regulation of renal *Cyp27b1* expression and that 1,25(OH)_2_D_3_ was a potent suppressor of this gene were established decades in the past (12, 13, 14). However, limited progress was made in understanding the mechanisms through which these two hormones as well as FGF23 function to control the expression of these genes until recently. Addressing this issue directly, we focused on the mouse as a model system, thereby overcoming the limitations imposed by experiments in cell culture, and together with improved technical approaches amendable to *in vivo* studies have provided new molecular insight into how PTH, FGF23, and 1,25(OH)_2_D_3_ control the expression of both *Cyp27b1* and *Cyp24a1* (10, 11, 15). Using chromatin immunoprecipitation sequencing (ChIP-Seq) analysis as a guide, together with CRISPR/Cas9-mediated genome deletion analysis *in vivo* to confirm function, we defined the genomic sites of action of each of these mineralotropic hormones across these two gene loci in the kidney (10, 11). In summary, we identified four regulatory regions located upstream of the *Cyp27b1* gene promoter; one (M1) was located within an intron of the adjacent *Mettl1* gene and three (M21 a–c) were collectively located within an extended intron in the more upstream *Mettl21b* (*Eef1akmt3*) gene (Fig. 1*B*). Deletion of the M1 region resulted in highly reduced expression of *Cyp27b1*, insensitivity to exogenous PTH, and a striking *Cyp27b1-*null-like phenotype in mice complete with highly elevated PTH, exceptionally low FGF23 levels, and highly compromised circulatory concentrations of 1,25(OH)_2_D_3_ (11). Bone loss was also evident. On the other hand, deletion of the M21 region resulted in a reduction in *Cyp27b1* expression, but only partial insensitivity to PTH, and was neither accompanied by elevated PTH levels nor changes in circulating 1,25(OH)_2_D_3_ and did not display a significant phenotype in the mouse (11). These results suggested that M1 represented a unique regulatory region controlled by PTH or that M1 was a hierarchical requirement for the activity of the three M21 regulatory regions. ChIP-Seq analysis revealed the presence of phospho-CREB at each of these four sites. However, determination of a functional association between PTH and CREB could not be established since CREB levels at these sites were unchanged following PTH treatment (10, 11). We also have reported modest activation of *Mettl21b* in response to PTH and interaction with these enhancers; however, *Mettl21b* global deletion did not have a skeletal or mineral phenotype (10). Additional regulatory studies revealed that 1,25(OH)_2_D_3_ induced vitamin D receptor (VDR) binding at each of these four sites and suppressed *Cyp27b1* expression. While FGF23 also suppressed *Cyp27b1* expression, the identity of a regulatory factor targeted by FGF23 signaling at the *Cyp27b1* gene remains unresolved. Of key importance, this complex regulatory module based upon other parameters was present only in the kidney, providing a molecular basis for the absence of regulated expression of *Cyp27b1* in nonrenal tissues by PTH, FGF23, and 1,25(OH)_2_D_3_. A summary of these activities is included in Figure 1*B*.

Interestingly, further studies revealed the presence of a similar module that mediated regulatory action by these three hormones in the *Cyp24a1* gene (15). In summary, while 1,25(OH)_2_D_3_ has long been known to regulate expression *via* a promoter–proximal (PP) element, we discovered an extended region located downstream of the gene that contained multiple regulatory sites that mediated suppression by PTH and induction by both FGF23 and 1,25(OH)_2_D_3_ as well (15, 16, 17). Deletion analysis in the mouse *in vivo* confirmed the functionality of these elements and defined two extended segments, a more gene-proximal segment that like *Cyp27b1* contained regulatory sites for PTH and FGF23 and bound CREB with a second more distal vitamin D responsive segment; deletion of this more proximal segment resulted in loss of response to both hormones but not to 1,25(OH)_2_D_3_, which induced VDR binding to a more distal downstream segment (15). Interestingly, like *Cyp27b1*, the PTH/FGF23-sensitive segment was present only in the kidney, providing a rationale for why in nonrenal tissues, *Cyp24a1* is resistant to PTH and FGF23 and not to 1,25(OH)_2_D_3_. Further deletion studies in mice, however, suggested that a PP regulatory site located near the *Cyp24a1* promoter played a predominant role in 1,25(OH)_2_D_3_ regulation in the kidney. The directional nature of *Cyp24a1* response to PTH, FGF23, and 1,25(OH)_2_D_3_ leads to a corresponding reciprocal change in *Cyp27b1* expression and vice versa *via* homeostatic mechanisms. A summary of these activities is included in Figure 1*C*.

The mechanism of PTH action in bone and kidney involve a triggering of multiple signal transduction pathways including the well-characterized PKA and PKC pathways; the former involving a secondary elevation in cellular cAMP, which leads to a complex G-protein involved signal transduction pathway that results in the activation of several transcription factors (TFs) including CREB at target genes (18, 19). Recent studies have now shown that PTH also modulates salt-inducible kinase (SIK) pathways in bone (20, 21, 22). PTH signaling leads to PKA-mediated SIK phosphorylation, a modification that inhibits cellular SIK activity *via* an allosteric mechanism. SIKs are widely expressed in numerous tissues including the kidney; PKA-dependent SIK inhibition causes the dephosphorylation-dependent cytoplasmic release of CREB-regulated transcription coactivators (CRTCs/TORCs) and their subsequent translocation into the nucleus (23, 24, 25). There, they interact directly with DNA-bound CREB and/or several other TFs and contribute along with CBP (CREB-binding protein)/p300, an additional CREB coactivator, to regulate gene expression. Recent investigations of the PTH-induced SIK pathway in bone has taken advantage of several pharmacologic SIK inhibitors such as YKL-05-099 and, more recently, SK-124 that cause the release of cytoplasmic CRTCs into the nucleus where they regulate the expression of specific PTH-sensitive genes that are involved in bone formation (22, 23, 25, 26, 27). Considerable insight has been gained regarding the molecular properties of both CBP and the CRTCs. CBP is a large multidomain-containing coregulator with multiple protein–protein interaction domains that may also facilitate chromatin reorganization as well as residual acetyltransferase activity at histone H3K27 (H3K27ac) (28, 29). The CRTCs, on the other hand, interact directly with CREB and potentially with specific DNA sequences but are not known to retain histone acetyltransferase activity (24). Although they are believed to stabilize DNA-bound CREB, likely additional roles in gene regulation for the CRTCs are not fully understood.

In the present report, we assess the impact of PTH, 1,25(OH)_2_D_3_, and FGF23 on hormone-regulated events that culminate in changes in the expression of *Cyp27b1* and *Cyp24a1* in the kidney. Importantly, we have focused this current report on only *Cyp27b1* and *Cyp24a1* despite the genome-wide datasets for all factors we have examined. A detailed genome-wide analysis will follow this article. We examine the temporal aspects of PTH induction and find these activities occur on a far more rapid timescale than we previously believed. These activities are accompanied by coactivator recruitment for activation (*Cyp27b1*) or coactivator withdrawal for suppression (*Cyp24a1*). Interestingly, direct pharmacologic SIK inhibition is able to induce CRTC2 coactivator recruitment; however, the overall activities are muted in comparison to PTH. There is limited overlap between these activities and the reciprocal 1,25(OH)_2_D_3_ and FGF23 suppression of *Cyp27b1* and activation of *Cyp24a1*. In both cases, these activities manifest themselves by direct changes to the factors responsible for transcription like RNA polymerase II (pol II) and many histone markers of activation. We suggest that multiple CREB modules together with alterations in CREB coactivators at these sites in both *Cyp27b1* and *Cyp24a1* genes drive appropriate selective reciprocal responses to PTH, 1,25(OH)_2_D_3_, and FGF23 regulation *in vivo*.

## Results

### Activation of CREB modules by PTH induces the expression of renal *Cyp27b1*

**Figure 2:**
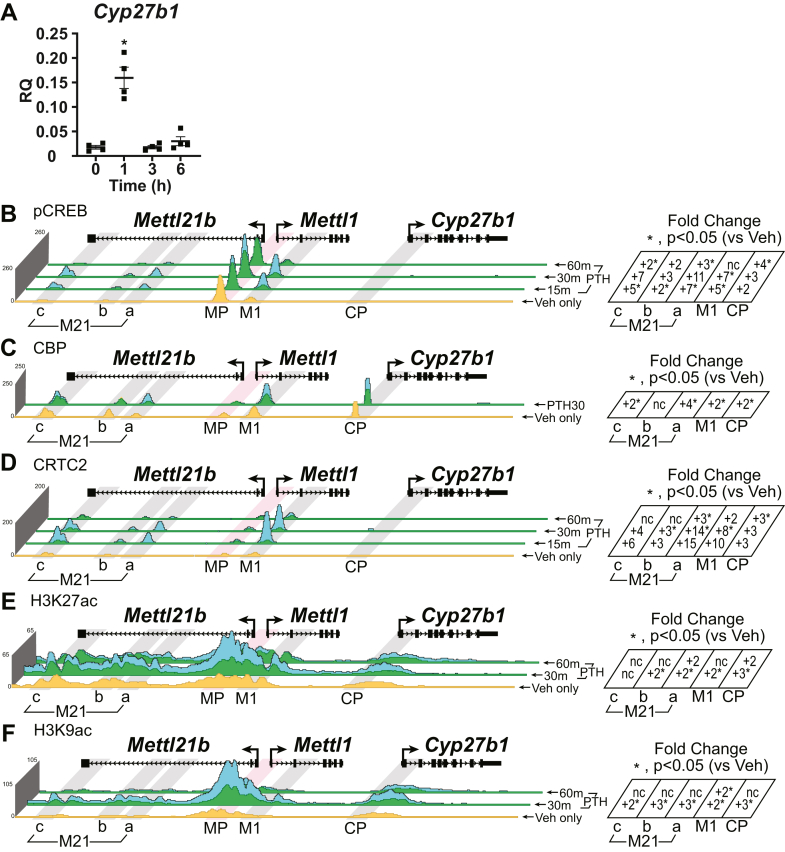
**Time course of PTH actions in mouse kidney near the *Cyp27b1* gene.***A*, gene expression in the kidney for *Cyp27b1* in 8- to 9-week-old WT C57BL/6 mice injected with 230 mg/kg bw PTH for 0, 1, 3, or 6 h. Data are displayed as relative quantitation (RQ, mean ± SEM) compared with *Gapdh*. n = 4 for each time point. One-way ANOVA with multiple comparison Tukey post test: ∗*p* < 0.05 time point *versus* 0 h. ChIP-Seq analysis near *Cyp27b1* for (*B*) pCREB, (*C*) CBP, (*D*) CRTC2, (*E*) H3K27ac, or (*F*) H3K9ac from WT mice injected with 230 mg/kg bw PTH for 0 (Veh only), 15, 30, or 60 min (n = 3). Overlaid triplicate and averaged ChIP-Seq tracks where Veh (0 h) are shown in *yellow*, treatments shown in *blue*, and overlapping data appear as *green*. Regions of interest are highlighted in *gray boxes* (M1, CP [*Cyp27b1* promoter], M21(a–c), tissue specific) or *red* (MP [*Mettl1/Mettl21b* promoters], nontissue specific). Genomic region displayed is chr10:126,469,260 to 126,490,800, and maximum height of tag sequence density for each data track indicated on the *Y*-axis (normalized to input and 10^7^ tags). Fold change table (*right*) was calculated for triplicate tag density in each peak region *versus* Veh. ∗*p* < 0.05 paired *t* test: treatment *versus* vehicle. nc, no change (<1.5-fold). bw, body weight; CBP, CREB-binding protein; ChIP-Seq, chromatin immunoprecipitation sequencing; CRTC, CREB-regulated transcription coactivator; H3K9ac, histone acetylation at histone H3 lysine 9; H3K27ac, histone acetylation at histone H3 lysine 27; pCREB, phosphorylated (p-133) CREB; PTH, parathyroid hormone.

PTH rapidly induces the expression of *Cyp27b1* in the kidney (Fig. 2*A*). Our previous studies revealed the presence of four regulatory units within intronic sequences in two genes located upstream of the *Cyp27b1* promoter (CP) that were occupied by phosphorylated (p-133) CREB (pCREB) (10, 11). We could not, however, confirm a potential relationship on the genome between PTH and pCREB because the recruitment of this factor was unaffected by hormone treatment at the chosen time point (1 h). An increase in histone H3 lysine 9 acetylation (H3K9ac) density was clearly evident at each of these four sites in the earlier study; however, indicating an increased activity of the locus in response to PTH (10, 11). Given the rapidity with which known exogenous PTH induces *Cyp27b1* gene expression, we initiated these current studies by conducting an abbreviated time course of response using ChIP-Seq analysis. For these studies, we will focus on results around *Cyp27b1* and *Cyp24a1* only, an article dedicated to the genome-wide analysis of these cofactors is in preparation. We injected mice with PTH and harvested the kidneys for ChIP and ChIP-Seq analysis after 0 (Veh), 15, 30, and 60 min to investigate this rapid action and then examined this genomic profile around the *Cyp27b1* locus with special focus on the previously identified kidney-specific enhancers M1 and M21 (a–c) for *Cyp27b1*, as well as near the CP (10, 11). We have also highlighted the *Mettl1/Mettl21b* promoter regions for comparison as these promoters are not kidney specific and are occupied in many other tissues of the mouse. We have displayed the vehicle as a stand-alone track for each track set. Each treatment condition (*blue*) listed is compared with an overlaid track against that vehicle track. Those regions where the treatment is greater than the vehicle (increase of occupancy), the track appears *blue*. Those where the treatment is less than the vehicle (loss of occupancy), *yellow* is displayed. Occupancies that overlap (treatment and vehicle) appear as *green*. We then performed statistics on the biological triplicate ChIP-Seq track read densities for each peak region and displayed them as fold change (FC) *versus* vehicle to the right of each track set. These FCs can be illuminating; however, one important caveat to the calculations based on sequencing read density is that there is no cutoff for minimal read density. Thus, extremely minor increases from an origin of low reads may appear as an appreciable increase by FC calculations. The unabbreviated values including the raw read density values are included in Table S1 and should be considered when evaluating each figure and data track. It is also important to note that 0 h (Veh) samples represent a mouse at “homeostasis,” that is this animal has normal and physiologic circulating levels of PTH (and many other hormones such as 1,25(OH)_2_D_3_ and intact FGF23 [iFGF23]). The outcome of this is genomic occupancy for TFs, coactivators, and histone modifications at a homeostatic state prior to hormone injection, which appear in our figures in the *yellow tracks*.

As can be seen in Figure 2*B*, PTH exerted a significant upregulation of pCREB abundance at each site across the two *Cyp27b1* regulatory submodules beginning as early as 15 m that peaked at 30 m and returned to below baseline by 1 h, the time point taken in the previous experiments. For example, the raw read counts at M1 were ∼57 that peaked maximally at ∼400 reads for PTH treatment for 30 min (Table S1). pCREB had very little binding to the CP at any time point (raw read values of ∼4 basally increased to ∼16, Table S1), consistent with our previous studies (10, 11). This upregulation of pCREB abundance supported our hypothesis that CREB was indeed one TF target of PTH at this gene. Given this upregulation, we then examined the effects of PTH on CBP, a primary coactivator associated with CREB activity at genes using the peak time point of 30 m. As can be seen in Figure 2*C*, PTH also induced an expected strong recruitment of CBP at these sites, compared with WT residual baseline. Finally, since recent studies have also suggested that PTH promotes an inhibition of SIKs that initiates the cytoplasmic to nuclear translocation of CRTC2 (20, 25), we also assessed whether CRTC2 was present at the regulatory regions of *Cyp27b1* and accumulated there in response to PTH. As can be seen in Figure 2*D*, CRTC2 was present basally as well as transiently and robustly recruited at each of the four regulatory sites (M1, M21a–c) bound by pCREB and CBP with a time course coincident with that of pCREB. These studies suggest that PTH strongly induced an active conformation within the multiple pCREB modules complete with at least two coactivators capable of activating transcription. Because ChIP-Seq analysis reveals only the presence of TFs at potentially active regions of DNA, these studies are not however, definitive for gene regulatory module activation. We therefore explored the ability of PTH to induce histone acetylation at histone H3 lysine 27 (H3K27ac, Fig. 2*E*) and lysine 9 (H3K9ac, Fig. 2*F*), the former a direct target of the histone acetylation properties of CBP. As can be seen in Figure 2, *E* and *F*, PTH induced a robust upregulation of H3K27ac and H3K9ac at each of the four sites, suggesting that this outcome, which is known to underlie increased gene transcript levels, is a direct consequence of the increased activity of the pCREB modules at the *Cyp27b1* locus. We conclude therefore that PTH induces a transcriptionally active agonist conformation of the pCREB modules capable of upregulating *Cyp27b1* expression.

### Inactivation of CREB modules by PTH downregulates the expression of renal *Cyp24a1*

**Figure 3:**
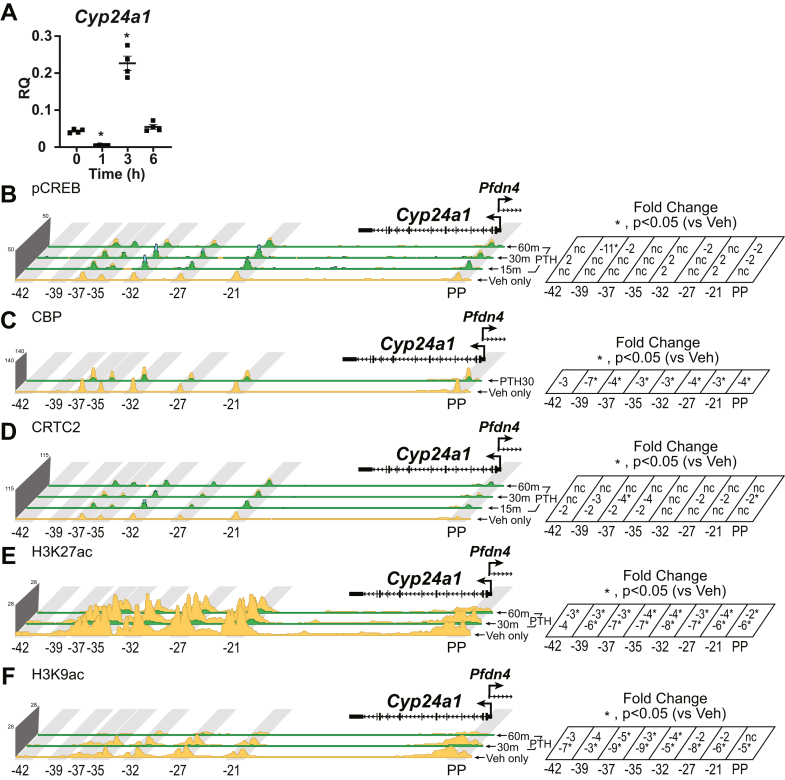
**Time course of PTH actions in mouse kidney near the *Cyp24a1* gene.***A*, gene expression in the kidney for *Cyp24a1* in 8− to 9–week-old WT C57BL/6 mice injected with 230 mg/kg bw PTH for 0, 1, 3, or 6 h. Data are displayed as relative quantitation (RQ, mean ± SEM) compared with *Gapdh*. n = 4 for each time point. One-way ANOVA with multiple comparison Tukey post-test: ∗*p* < 0.05 time point *versus* 0 h. ChIP-Seq analysis near *Cyp24a1* for (*B*) pCREB, (*C*) CBP, (*D*) CRTC2, (*E*) H3K27ac, or (*F*) H3K9ac from WT mice injected with 230 mg/kg bw PTH for 0 (Veh only), 15, 30, or 60 min (n = 3). Additional details as for Figure 2. Genomic region displayed is chr2: 170,278,379 to 170,324,235. bw, body weight; CBP, CREB-binding protein; ChIP-Seq, chromatin immunoprecipitation sequencing; CRTC, CREB-regulated transcription coactivator; H3K9ac, histone acetylation at histone H3 lysine 9; H3K27ac, histone acetylation at histone H3 lysine 27; pCREB, phosphorylated (p-133) CREB; PTH, parathyroid hormone.

Our previous studies of the renal *Cyp24a1* gene revealed the presence of pCREB at multiple regulatory sites located near the *Cyp24a1* promoter and particularly in a large intergenic region downstream of the gene (15). We therefore used ChIP-Seq analysis to explore the temporal abundance of pCREB at these sites in the *Cyp24a1* locus and to assess the ability of PTH to regulate CBP and CRTC2 binding as well. We had previously defined a set of enhancer regions downstream of the *Cyp24a1* gene (−21 through −42 kb) as being essential for the kidney-specific PTH and FGF23 regulation of *Cyp24a1* (15, 17). There, we termed the collection of enhancers from -21 to -32 kb as the DownStream 1 region and the −35 to −37 kb as the DownStream 2 region (15); for clarity, we will refer to each peak by its distance in kilobase from the transcriptional start site with associated FCs shown to the right of each track set. The PP region is vital for 1,25(OH)_2_D_3_-mediated actions throughout all tissues, including the kidney (15). As can be seen in Figure 3*A*, PTH strongly suppressed *Cyp24a1* expression from residual expression levels, although because of the rapid and transient lifetime of PTH administered exogenously intraperitoneally, there was a rapid recovery and corresponding increase in *Cyp24a1* expression within 3 h because of increasing 1,25(OH)_2_D_3_ concentrations, an effect that occurs opposite that for *Cyp27b1* as well. As seen in Figure 3*B*, ChIP-Seq analysis revealed that PTH had little effect on pCREB or CRTC2 (Fig. 3*D*) at each of the sites downstream (−21 to −42 kb) region. Importantly, however, PTH caused a dismissal of residual CBP (Fig. 3*C*) across all downstream (−21 to −42 kb) regions. These actions of PTH corresponded temporally to a strong decrease in residual H3K27ac (Fig. 3*E*) and H3K9ac (Fig. 3*F*) and an elimination of *Cyp24a1* transcripts. These results indicate that while complex with respect to the relative presence of pCREB and CRTC2 as well as reduced CBP at multiple sites across the gene, PTH appears to induce a conformation with the CREB modules that is inhibitory to productive transcription of *Cyp24a1*. The relative role of the PP enhancer at the *Cyp24a1* gene is not entirely clear in these studies.

### Pharmacologic inhibition of the SIKs by YKL-05-099 and SK-124 induces CRTC2 binding and a subset of PTH-like activities at the *Cyp27b1* and *Cyp24a1* genes

**Figure 4:**
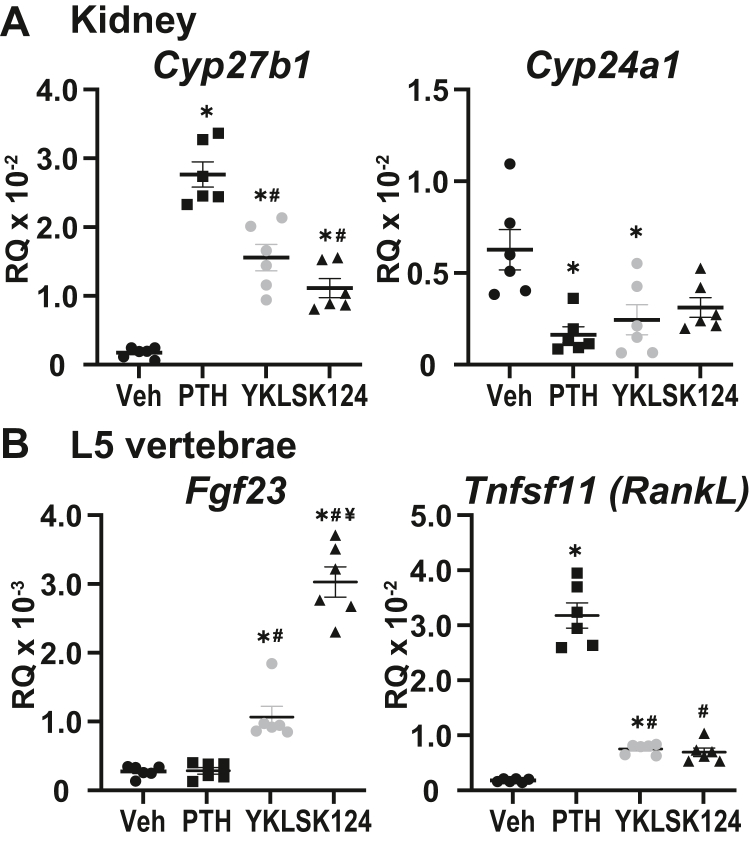
**SIK inhibition increases *Cyp27b1* and decreases *Cyp24a1* expression.***A*, gene expression of *Cyp27b1* (*left*) and *Cyp24a1* (*right*) in the kidney and *B*, *Fgf23* (*left*) and *Tnfsf11* (*right*) in the L5 vertebrae from WT mice treated with vehicle (Veh, 3 h), 230 mg/kg PTH (1 h), 30 mg/kg YKL-05-099 (3 h), or 40 mg/kg SK-124 (3 h). Data are displayed as relative quantitation (RQ, mean ± SEM) compared with *Gapdh*. n = 6 for each treatment. One-way ANOVA with multiple comparison Tukey post-test: ∗*p* < 0.05 treatment *versus* Veh. #*p* < 0.05 treatment *versus* PTH. parathyroid hormone; SIK, salt-inducible kinase.

With the involvement of CRTC2 on the genomic locus, presumably contributing to the activation of *Cyp27b1*, we sought to dissect this mechanism further using selective agents that promote CRTC2 nuclear translocation without activating PKA (20, 22, 27, 30). The SIK inhibitors are able to liberate CRTC2 from SIK phosphorylation, which allows CRTC2 to translocate to the nucleus to aid in the activation of transcription (24, 25). We were able to test two such inhibitors and compare their overall impact on gene expression in the mouse kidney and the L5 vertebrae. The structurally unrelated SIK inhibitors, YKL-05-099 and SK-124, were recently investigated for their ability to activate transcription in skeletal cells (20, 22, 26, 31). As seen in Figure 4*A*, we injected mice with PTH, YKL-05-099, and SK-124 and examined the effect on gene expression of *Cyp27b1* and *Cyp24a1* in the mouse kidney. We started with the published dose and time (20, 26, 31) of 30 mg/kg YKL-05-099 and 40 mg/kg SK-124 for 3 h and compared these with 230 mg/kg PTH for 1 h. We find that at these doses and time, the YKL-05-099 is half and SK-124 a third of the maximal expression on *Cyp27b1* treated with PTH; however, both drugs were able to induce *Cyp27b1* expression. *Cyp24a1* expression was suppressed by YKL-05-099 almost as effectively as PTH; however, the SK-124 failed to reach significance in its suppression of *Cyp24a1* in this experiment, at this time point. To compare with the previous skeletal cell studies (20, 22, 26), we also examined gene expression in the L5 vertebrae with the target genes of *Fgf23* and *Tnfsf11* (*RankL*). Both *Fgf23* and *Tnfsf11* were increased by these SIK inhibitors, in fact, the *Fgf23* expression was greatly increased with SK-124. These results led us to next investigate the dose and time optimization for both YKL-05-099 and SK-124 as well as whether they were able to increase CRTC2 genomic occupancy to help drive transcription.

**Figure 5:**
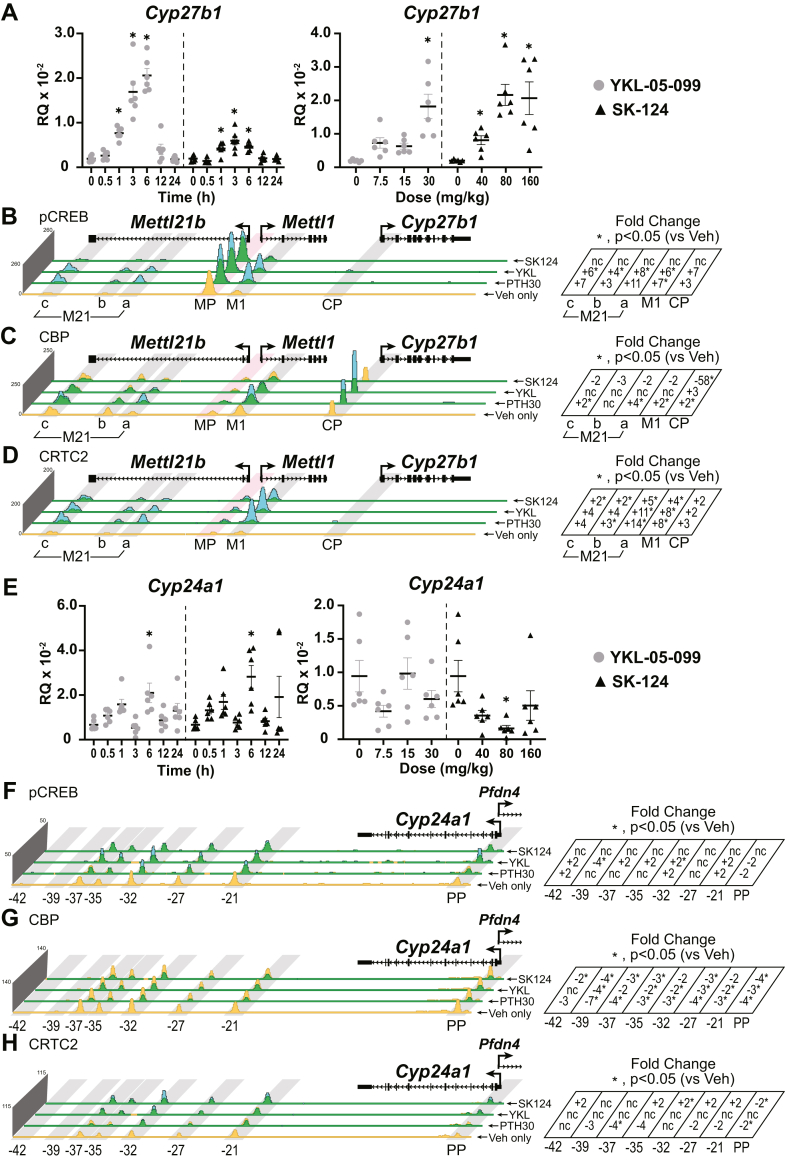
**SIK inhibitors YKL-05-099 and SK-124 have PTH-like actions on the genome near *Cyp27b1* and *Cyp24a1*.***A*, gene expression in the kidney for *Cyp27b1* after WT mouse treatment with 30 mg/kg bw YKL-05-099 or 40 mg/kg bw SK-124 for 0, 0.5, 1, 3, 6, 12, 24 h (*left*) or 3 h YKL-05-099 at 7.5, 15, and 30 mg/kg bw and SK-124 at 40, 80, and 120 mg/kg bw (*right*). Data are displayed as relative quantitation (RQ, mean ± SEM) compared with *Gapdh*. n = 6 for time point. One-way ANOVA with multiple comparison Tukey post-test: ∗*p* < 0.05 time point or treatment *versus* 0 h. ChIP-Seq analysis near *Cyp27b1* for (*B*) pCREB, (*C*) CBP, and (*D*) CRTC2 from WT mice injected with 230 mg/kg bw PTH for 30 min (PTH30), 30 mg/kg bw YKL-05-099 for 1 h, or 40 mg/kg bw SK-124. PTH30 data tracks are same as for Figure 2. Additional details as for Figure 2. Genomic region displayed is chr10: 126,469,260 to 126,490,800. *E*, *Cyp24a1* expression in the mouse kidney after treatments described in *A*. ChIP-Seq analysis near *Cyp24a1* for (*F*) pCREB, (*G*) CBP, and (*H*) CRTC2 as described above in *B*–*D*. PTH30 data tracks same as for Figure 3. Additional details as for Figure 2. Genomic region displayed is chr2: 170,278,379 to 170,324,235. bw, body weight; CBP, CREB-binding protein; ChIP-Seq, chromatin immunoprecipitation sequencing; CRTC, CREB-regulated transcription coactivator; pCREB, phosphorylated (p-133) CREB; PTH, parathyroid hormone; SIK, salt-inducible kinase.

We conducted a time course and dose response for *in vivo* YKL-05-099 and SK-124 treatment in kidney (Fig. 5*A*). We found that the maximal activity of a 30 mg/kg dose of YKL-05-099 peaked between 3 and 6 h as these points were not statistically different from each other, and the SK-124 40 mg/ml dose peaked at 3 h. The maximal activity of YKL-05-099 and SK-124 at 3 h was more delayed or prolonged compared with PTH (1 h, Fig. 2*A*) because of differences in pharmacokinetics between these small-molecule kinase inhibitors and the peptide hormone (27). These data demonstrate PTH-like effects of SIK inhibitors in kidney similar to those previously reported in bone (22).

We then examined the ability of both YKL-05-099 and SK-124 to induce genomic binding around the *Cyp27b1* gene locus in a similar manner to PTH. Here, we compared the 30 mg/kg dose of YKL-05-099 and 40 mg/ml dose of SK-124 at 1 h to the PTH maximal dose at 30 min. These times were in line with our previous times used for 1,25(OH)_2_D_3_ activation of the VDR binding to the genome (1 h ChIP-Seq, 6 h peak gene expression) (10, 11, 15). In Figure 5*B*, we found that YKL-05-099 was able to induce pCREB binding similarly to PTH, whereas SK-124 did not enrich pCREB binding at our M1 or M21(a–c) enhancers. pCREB bound sixfold to eightfold over vehicle at the M1 and M21(a–c) enhancers with YKL-05-099. Interestingly, CBP occupancy increased at the CP region (Fig. 5*C*) with PTH and YKL-05-099 treatments, but CBP was dismissed from the CP by SK-124 (−58 FC). The M1 and M21(a–c) enhancers were unchanged from their vehicle levels. In contrast, CRTC2 was recruited to the M1 and M21(a–c) enhancers with both YKL-05-099 and SK-124 similar to PTH (Fig. 5*D*). In Figure 5, *E*–*H*, we examined the YKL-05-099 and SK-124 activities around *Cyp24a1* and found that the behavior of YKL-05-099 and SK-124 was very similar to PTH with a loss of CBP and unchanged recruitment of CRTC2. The pCREB, though, appeared to be increased twofold to fourfold across the PP and the downstream enhancers (-21 to -37kb). The gene expression of *Cyp24a1* in Figure 5*E* was far more variable than that seen for *Cyp27b1* expression.

**Figure 6:**
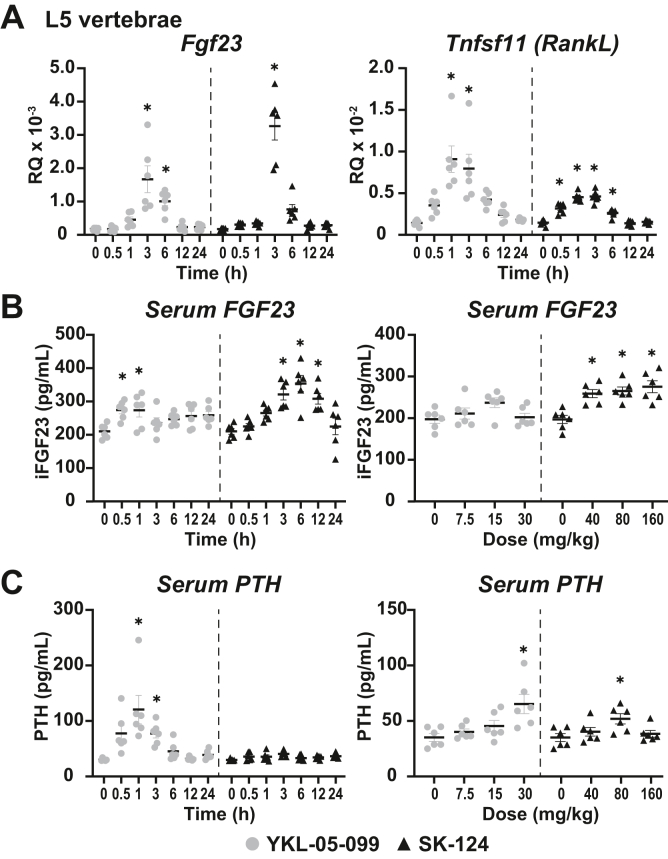
**SIK inhibitors elevate serum PTH and/or serum iFGF23.***A*, gene expression in the L5 vertebrae for *Fgf23* and *Tnfsf11* after WT mouse treatment with 30 mg/kg bw YKL-05-099 or 40 mg/kg bw SK-124 for 0, 0.5, 1, 3, 6, 12, and 24 h. Serum measurements for (*B*) iFGF23 (pg/ml) and (*C*) PTH (pg/ml) for same dose and time as indicated for Figure 5. n = 6 for time point. One-way ANOVA with multiple comparison Tukey post-test: ∗*p* < 0.05 time point or treatment *versus* 0 h. bw, body weight; iFGF23, intact fibroblast growth factor 23; PTH, parathyroid hormone; SIK, salt-inducible kinase.

We then examined the serum levels of PTH, iFGF23, Ca, and phosphate in the animals treated with both compounds. Similar to the results in Figure 4, we found a time-dependent increase of the skeletal genes *Fgf23* and *Tnfsf11* after treatment with YKL-05-099 and SK-124 (Fig. 6*A*). Interestingly, *Fgf23*, the negative regulator of *Cyp27b1*, was upregulated by both YKL-05-099 and SK-124. This in turn raised the serum iFGF23 significantly with both YKL-05-099 and SK-124 (Fig. 6*B*). The serum PTH was significantly increased at the 30 mg/kg dose of YKL-05-099 at 1 and 3 h postinjection (Fig. 6*C*); however, SK-124 at the 40 mg/kg dose did not affect serum PTH levels through 24 h. The serum PTH increases precede the maximal gene expression for YKL-05-099 that was observed (Fig. 5*A*). It would appear as though the observed activity of YKL-05-099 on *Cyp27b1* and *Cyp24a1* can be in part attributed to the increase of serum PTH. However, the maximal increase of iFGF23 by the SK-124 compound does so only after maximal gene expression of *Cyp27b1* (Fig. 5*A*). The *Cyp27b1* gene expression increases by SK-124 appear to be dampened by the increasing iFGF23 levels in the serum. The serum Ca and phosphate were not significantly altered (data not shown), which may indicate activities unrelated to Ca and P in the regulation of PTH and iFGF23 release from the parathyroid glands and skeleton, respectively, and these mechanisms are currently under investigation.

### 1,25(OH)_2_D_3_ and FGF23 suppress the activity of the pCREB module in the *Cyp27b1* gene

**Figure 7:**
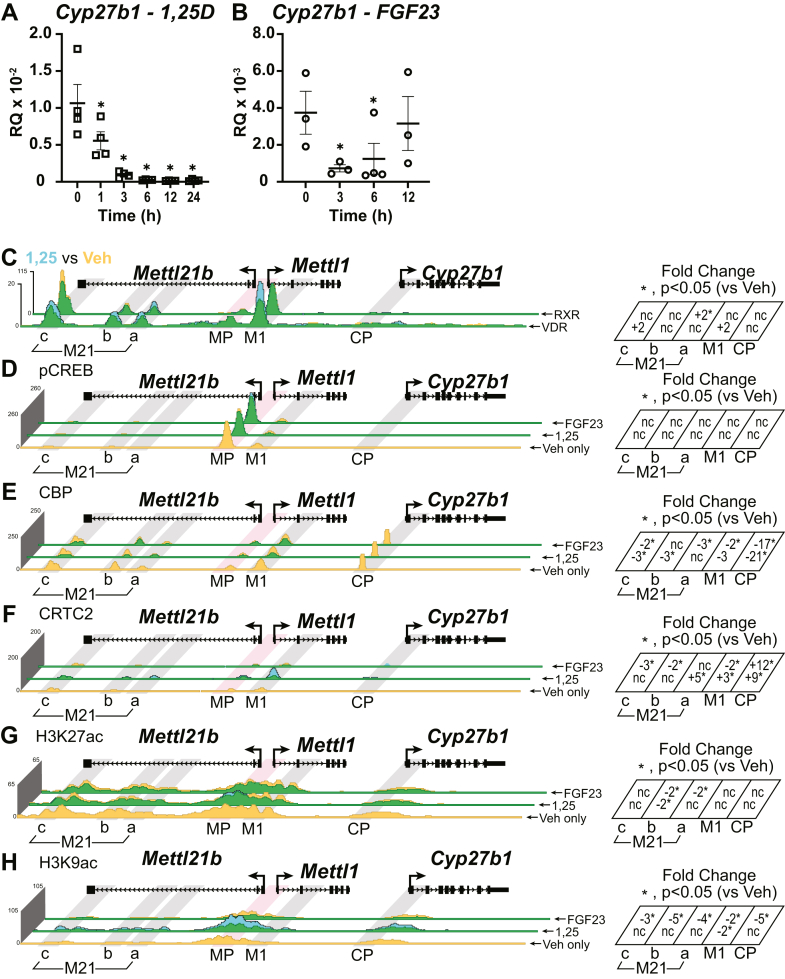
**Activities of 1,25(OH)**_**2**_**D**_**3**_**and FGF23 at *Cyp27b1*.** Gene expression of *Cyp27b1* after time course with (*A*) 10 mg/kg bw 1,25(OH)_2_D_3_ at 0, 1, 3, 6, 12, and 24 h (n = 4) and (*B*) 50 mg/kg bw FGF23 at 0, 3, 6, and 12 h (n = 3–4) from our previous publication (10). ChIP-Seq analysis near *Cyp27b1* for (*C*) VDR and RXR (1,25(OH)_2_D_3_*versus* Veh only), (*D*) pCREB, (*E*) CBP, (*F*) CRTC2, (*G*) H3K27ac, and (*H*) H3K9ac from WT mice injected with 10 mg/kg bw 1,25(OH)_2_D_3_ or 50 mg/kg bw FGF23 (1 h). Additional details as for Figure 2. Genomic region displayed is chr10: 126,469,260 to 126,490,800. bw, body weight; CBP, CREB-binding protein; ChIP-Seq, chromatin immunoprecipitation sequencing; CRTC2, CREB-regulated transcription coactivator 2; FGF23, fibroblast growth factor 23; H3K9ac, histone acetylation at histone H3 lysine 9; H3K27ac, histone acetylation at histone H3 lysine 27; 1,25(OH)_2_D_3_, 1,25-dihydroxyvitamin D_3_; pCREB, phosphorylated (p-133) CREB; RXR, retinoid X receptor; VDR, vitamin D receptor.

Induction of *Cyp27b1* and suppression of *Cyp24a1* by PTH is only part of the hormonal control that occurs in the kidney for regulation of vitamin D metabolism. Increased circulating 1,25(OH)_2_D_3_ suppresses *Cyp27b1* expression in a feedback control mechanism and strongly induces *Cyp24a1* to facilitate 1,25(OH)_2_D_3_ clearance. Serum iFGF23 can increase in response to elevated serum phosphate levels or rising 1,25(OH)_2_D_3_ levels or by injection of iFGF23. Regardless of the cause of the rise, serum iFGF23 is a potent suppressor of *Cyp27b1* expression and moderate inducer of *Cyp24a1*. As can be seen in Figure 7*A*, *Cyp27b1* expression is decreased by 1,25(OH)_2_D_3_ injection over time and remains suppressed throughout the 24 h time point (10). A single iFGF23 injection displays a more transient suppression of *Cyp27b1* that peaks at 3 h and returns to baseline by 12 h (Fig. 7*B*) (10). It is important to note that serum iFGF23 levels increase and remain elevated after 1,25(OH)_2_D_3_ injection, which likely prolongs the suppression of 1,25(OH)_2_D_3_ through secondary mechanisms (10).

**Figure 8:**
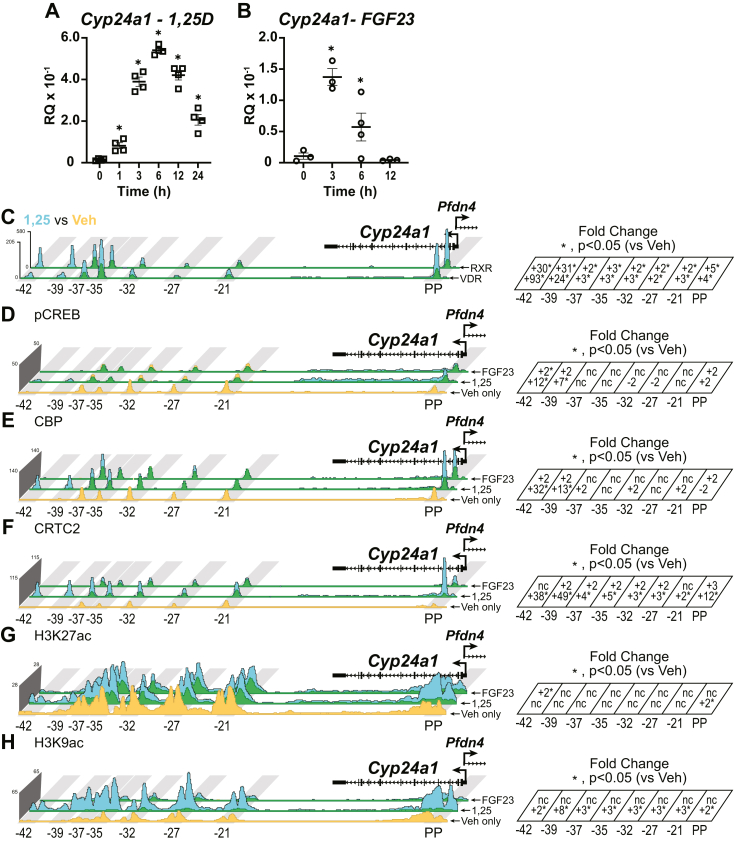
**Activities of 1,25(OH)**_**2**_**D**_**3**_**and FGF23 at *Cyp24a1*.** Gene expression of *Cyp24a1* after time course with (*A*) 10 mg/kg bw 1,25(OH)_2_D_3_ at 0, 1, 3, 6, 12, and 24 h (n = 4) and (*B*) 50 mg/kg bw FGF23 at 0, 3, 6, and 12 h (n = 3–4) from our previous publication (10). ChIP-Seq analysis near *Cyp24a1* for (*C*) VDR and RXR (1,25(OH)_2_D_3_*versus* Veh only), (*D*) pCREB, (*E*) CBP, (*F*) CRTC2, (*G*) H3K27ac, and (*H*) H3K9ac from WT mice injected with 10 mg/kg bw 1,25(OH)_2_D_3_ or 50 mg/kg bw FGF23 (1 h). Additional details as for Figure 2. Genomic region displayed is chr2: 170,278,379 to 170,324,235. bw, body weight; CBP, CREB-binding protein; ChIP-Seq, chromatin immunoprecipitation sequencing; CRTC2, CREB-regulated transcription coactivator 2; FGF23, fibroblast growth factor 23; H3K9ac, histone acetylation at histone H3 lysine 9; H3K27ac, histone acetylation at histone H3 lysine 27; 1,25(OH)_2_D_3_, 1,25-dihydroxyvitamin D_3_; pCREB, phosphorylated (p-133) CREB; RXR, retinoid X receptor; VDR, vitamin D receptor.

We have established that after a 10 mg/kg dose of 1,25(OH)_2_D_3_ for 1 h, VDR is recruited to the M1 and M21(a–c) enhancers in our previous work, and elimination of both M1 and M21(a–c) leads to a loss of 1,25(OH)_2_D_3_-mediated suppression of *Cyp27b1* (10, 11). In Figure 7*C*, we revisit that experiment with new VDR-binding data and extend our observation to include the VDR-binding partner retinoid X receptor (RXR). VDR occupancy increased between twofold and fivefold across these enhancers. RXR, on the other hand, was bound to DNA in the homeostatic condition (Veh, *yellow*), and this occupancy was not increased with 1,25(OH)_2_D_3_ treatment outside the M21a enhancer. This contrasts with other VDR enhancers throughout the genome where RXR occupancy also rises together with VDR, for example, around *Cyp24a1* (Fig. 8*C*). This may lead to a different binding modality for VDR; however, we have no evidence for anything outside a classical VDRE (hexameric DR3 element) as this sequence contains several typical VDREs that when eliminated did lessen VDR response (10). An increase of VDR occupancy during suppression of *Cyp27b1* remains a curious mechanistic question, and to understand more of this response, we turned to coactivators and histone modifications.

We found that in Figure 7*D*, the pCREB occupancy was not changed with 1,25(OH)_2_D_3_ treatment indicating that there may be no interplay between these TFs. CBP in Figure 7*E* demonstrated a loss of CBP binding across the enhancers hinting at a loss of histone acetylation, thus decreasing transcription. CRTC2 though (Fig. 7*F*) did increase at the M1, M21c, and the CP with the treatment of 1,25(OH)_2_D_3_. CRTC2 binding at CP was extremely low, so small increases in CRTC2 showed a large FC. FGF23 was only able to change CRTC2 binding at CP and was suppressed at the M1 and M21(a–c) enhancers. For histone modifications, we found that similar to CBP, the CBP target of H3K27ac (Fig. 7*G*) was also decreased. This was also true for FGF23 treatment and the H3K9ac response (Fig. 7*H*); however, 1,25(OH)_2_D_3_ increased H3K9ac near the *Mettl1/Mettl21b* promoter region and across the M21 enhancers albeit the FC calculations yielded “no change.” These calculations at enhancers where TFs are bound are somewhat problematic as the histone markers vacate the TF binding sites through nucleosome repositioning and create a valley, but nonetheless, the ChIP-Seq profiles qualitatively relate to histone marker increases and decreases.

### 1,25(OH)_2_D_3_ and FGF23 induce the activity of the pCREB module in the *Cyp24a1* gene

1,25(OH)_2_D_3_ is a strong activator of *Cyp24a1*, whereas FGF23 is a moderate activator of *Cyp24a1*. As previously observed, and shown in Figure 8*A*, 1,25(OH)_2_D_3_ increases *Cyp24a1* expression rapidly and peaks at 6 h (10). A single injection of FGF23 peaks at 3 h and rapidly returns to baseline by 12 h (Fig. 8*B*). As we observed with *Cyp27b1* activity in Figure 7, *A* and *B*, 1,25(OH)_2_D_3_ increases serum iFGF23, which likely aids in the prolonged activity of *Cyp24a1* increases as it did with *Cyp27b1* suppression. When we examined the VDR and RXR binding around the *Cyp24a1* locus, we found that VDR and RXR occupancy was strongly increased 3- to 10-fold at the PP (*Cyp24a1* PP) as well as the downstream enhancers (−21 to −42 kb). These peaks, in fact, are some of the largest for VDR and RXR occupancy in the entire genome. As we saw with *Cyp27b1* (Fig. 7), pCREB (Fig. 8*D*) was not drastically changed at the downstream enhancers; however, there was an increase of pCREB along the gene body and at the PP. CBP occupancy (Fig. 8*E*) was increased at some of the downstream enhancers and the PP by 1,25(OH)_2_D_3_. CRTC2 was also increased at the enhancers and the PP (Fig. 8*F*), much like they were around *Cyp27b1*. These coactivator changes did have an impact on the histone acetylation profiles, with both 1,25(OH)_2_D_3_ and FGF23 increasing the H3K27ac and H3K9ac markers (Fig. 8, *G* and *H*). The activation profile for *Cyp24a1* with 1,25(OH)_2_D_3_ and FGF23 did not utilize the downstream (−21 to −42 kb) enhancers for pCREB; however, it did appear that those enhancers, along with the PP, were involved in the increased occupancy of the coactivators and the histone markers.

### SRC1 and SRC3 are recruited for modulation of both *Cyp27b1* and *Cyp24a1* expression

**Figure 9:**
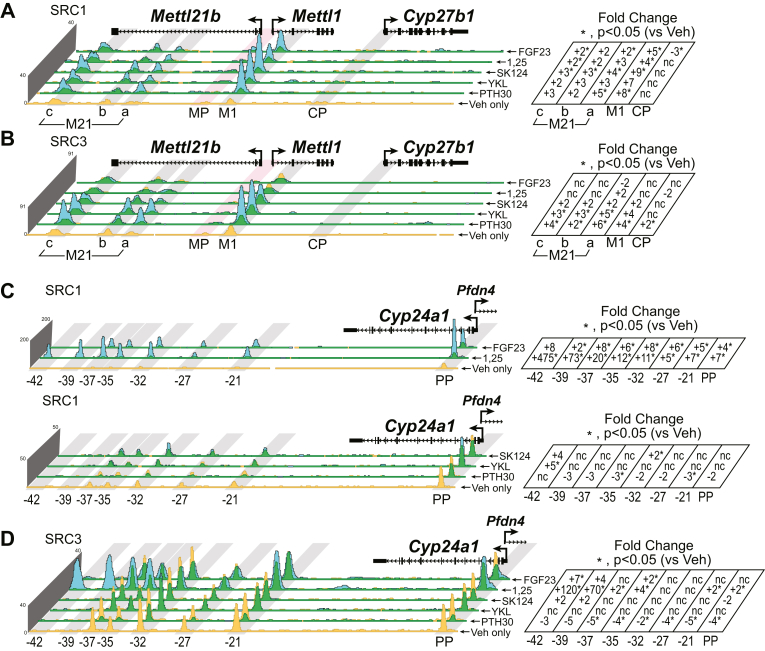
**SRC1 and SRC3 recruitment at *Cyp27b1* and *Cyp24a1*.** ChIP-Seq analysis near *Cyp27b1* for (*A*) SRC1, (*B*) SRC3 and *Cyp24a1*, (*C*) SRC1, (*D*) SRC3 from WT mice injected with 230 mg/kg bw PTH (30 min, PTH30), 30 mg/kg bw YKL-05-099 (1 h), 40 mg/kg SK-124 (1 h), 10 mg/kg bw 1,25(OH)_2_D_3_ (1 h), or 50 mg/kg bw FGF23 (1 h). Additional details as for Figure 2. Genomic regions displayed are chr10: 126,469,260 to 126,490,800 and chr2: 170,278,379 to 170,324,235. bw, body weight; ChIP-Seq, chromatin immunoprecipitation sequencing; 1,25(OH)_2_D_3,_ 1,25-dihydroxyvitamin D_3_; PTH, parathyroid hormone.

CRTC2 and CBP are not the only coactivators implicated with pCREB and VDR actions on the genome. SRC1 and SRC3 are a few examples of other known coactivators to be associated with both pCREB and VDR (32). While a powerful technique, ChIP-Seq is limited in its scope to the antibody used for immunoprecipitation. Therefore, while we see CRTC2 and CBP as bound coactivators, there may be dozen more factors involved in the regulation of genes responsive to PTH, FGF23, and 1,25(OH)_2_D_3_. To this end, we also examined the recruitment of SRC1 and SRC3 to the *Cyp27b1* and *Cyp24a1* loci as seen in Figure 9. In Figure 9*A*, we found that SRC1 was recruited to the M1 and M21(a–c) enhancers with all treatments. This recruitment was strongest with SK-124, PTH, and YKL-05-099; however 1,25(OH)_2_D_3_ and FGF23 both recruited SRC1 despite the overall net effect on gene expression being suppression. SRC3 at *Cyp27b1* showed recruitment with PTH, YKL-05-099, and SK-124; however, now 1,25(OH)_2_D_3_ and FGF23 show a reduction (or no change) in SRC3 occupancy across the M1 and M21(a–c) enhancers. In *Cyp24a1* occupancy (Fig. 9, *C* and *D*), SRC1 is strongly recruited to all enhancers with both 1,25(OH)_2_D_3_ and FGF23 (activators of *Cyp24a1* expression, Fig. 9*C*, *upper tracks*); however, there is a slight reduction or no change in SRC1 recruitment with PTH, YKL-05-099, and SK-124 (suppressors of *Cyp24a1* expression, Fig. 9*C*, *lower tracks*, scale is fourfold lower than upper tracks). SRC3 was only recruited to the 1,25(OH)_2_D_3_-treated *Cyp24a1* locus with a focus on the more distal regions (-35 to -42 kb), which are those known to recruit and activate *Cyp24a1* through VDR. PTH, YKL-05-099, and SK-124 had very little effect on the recruitment of SRC3 to *Cyp24a1*. From these observations, we can conclude that SRC1, and modestly SRC3, may be contributing to the genomic activation of *Cyp27b1* and *Cyp24a1* genes.

### The opposing actions of PTH by both 1,25(OH)_2_D_3_ and FGF23 are mediated *via* changes in CREB module components at Cyp27b1 and Cyp24a1 and show regulatory dominance

These unexpected activities of pCREB recruitment in *Cyp24a1* and the involvement of CRTC2 in 1,25(OH)_2_D_3_ activation led us to question the interplay, if any, between these opposing activity profiles. In particular, the increase of genomic occupancy by CRTC2 in the presence of 1,25(OH)_2_D_3_ and FGF23 was puzzling. To investigate these perplexing mechanisms, we looked at cotreatments of hormones and the consequence to genomic occupancy in Figs. S1 and S2. We did not investigate gene expression since the peak activation times were too disparate: 1 h for PTH maximal activity, 3 h for FGF23, and 6 h for 1,25(OH)_2_D_3_. However, maximal genomic actions were closer in timing: PTH at 30 min and FGF23, 1,25(OH)_2_D_3_, and YKL-05-099 at 1 h. A time line of treatments and doses are shown in Fig. S1*A*. We then looked at the genomic occupancy of pCREB, CBP, and CRTC2 in these cotreatment conditions around the *Cyp27b1* genomic locus. As can be seen in Fig. S1*B*, pCREB occupancy is decreased with the addition of the suppressors FGF23 and 1,25(OH)_2_D_3_ compared with the PTH and YKL-05-099 specific tracks. This is most noticeable at the M1 enhancer region where the activity decreases from sixfold and sevenfold, to twofold for PTH and YKL-05-099, respectively. In Fig. S1*C*, the CBP data follow a similar pattern to pCREB, the activities of PTH and YKL-05-099 were muted by the addition of FGF23 or 1,25(OH)_2_D_3_. In both pCREB and CBP, 1,25(OH)_2_D_3_ or FGF23 did not change the genomic occupancy around *Cyp27b1* in the absence of PTH or YKL-05-099. However, CRTC2 did increase in response to 1,25(OH)_2_D_3_ (Fig. 7*F*) at the M1 and CP, and FGF23 did increase CRTC2 occupancy at CP. When 1,25(OH)_2_D_3_ is treated with PTH and YKL-05-099 (Fig. S1*D*), CRTC2 occupancy is unchanged from the PTH and YKL-05-099 alone. Intriguingly, FGF23 cotreatment with PTH and YKL-05-099 increased CRTC2 genomic occupancy from eightfold to 14-fold. These results are interesting considering that FGF23 alone had a very minimal effect on CRTC2.

*Cyp24a1* genomic occupancy (Fig. S2) was also affected by the cotreatments of 1,25(OH)_2_D_3_ or FGF23 with PTH or YKL-05-099. pCREB (Fig. S2*A*) showed an unexpected synergistic effect of 1,25(OH)_2_D_3_ with PTH or YKL-05-099, most profound not only at the PP but also at the downstream enhancers. This increase was far greater than 1,25(OH)_2_D_3_, PTH, or YKL-05-099 alone. Considering the opposing nature of these signals, these results, like that seen with CRTC2 at *Cyp27b1*, are difficult to understand. CBP activity was dominated by the activator of 1,25(OH)_2_D_3_ and was unaffected by the cotreatment of PTH and YKL-05-099 (Fig. S2*B*). CBP after FGF23 and PTH or YKL-05-099 cotreatment on the other hand showed a decreased activity. This fits with expected activity as PTH and YKL-05-099 alone decreased CBP occupancy. CRTC2 occupancy (Fig. S2*C*) after 1,25(OH)_2_D_3_ cotreatment with PTH or YKL-05-099 was largely unaffected. The apparent FGF23-induced CRTC2 increase at the PP was decreased only with PTH, whereas the YKL-05-099 was unaffected. Overall, the genomic effects of the cotreatments are very complex. A complete cataloging of an extended profile of spatial events may be needed to understand this complicated interplay; however, it does appear that in these conditions, the activation and suppression complexes do have a relationship with respect to pCREB and the coactivators of CBP and CRTC2.

### PTH, FGF23, 1,25(OH)_2_D_3_, and SIK inhibition transmit profound transcriptional changes on the genome

**Figure 10:**
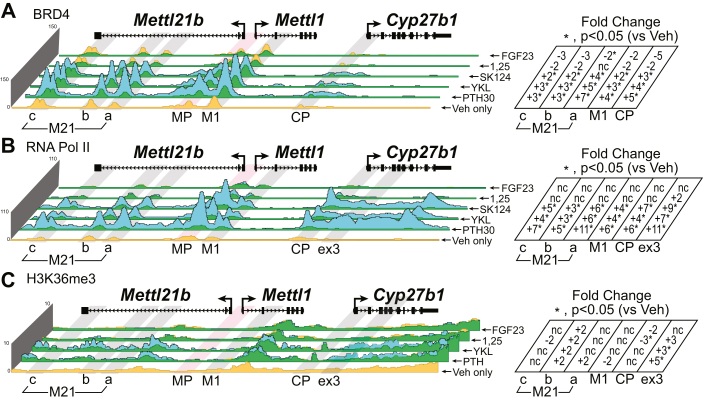
**Transcriptional implications of treatments at *Cyp27b1*.** ChIP-Seq analysis near *Cyp27b1* for (*A*) BRD4, (*B*) RNA polymerase II, and (*C*) H3K36me3 from WT mice injected with 230 mg/kg bw PTH (30 min, PTH30), 30 mg/kg bw YKL-05 to 099 (1 h), 40 mg/kg bw SK-124 (1 h), 10 mg/kg bw 1,25(OH)_2_D_3_ (1 h), or 50 mg/kg bw FGF23 (1 h). Additional details as for Figure 2. Genomic region displayed is chr10: 126,469,260 to 126,490,800. bw, body weight; ChIP-Seq, chromatin immunoprecipitation; FGF23, fibroblast growth factor 23; 1,25(OH)_2_D_3_, 1,25-dihydroxyvitamin D_3_; PTH, parathyroid hormone.

**Figure 11:**
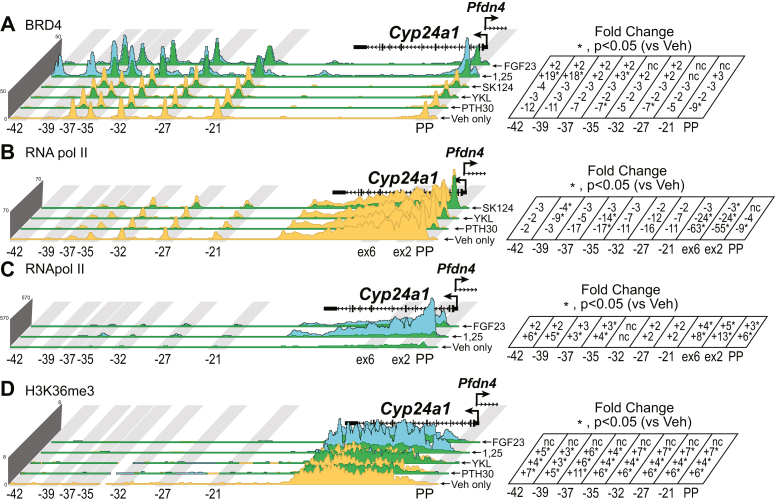
**Transcriptional implications of treatments at *Cyp24a1*.** ChIP-Seq analysis near *Cyp24a1* for (*A*) BRD4, (*B*) RNA polymerase II (scaled to 70), (*C*) RNA polymerase II (scaled to 570), and (*D*) H3K36me3 from WT mice injected with 230 mg/kg bw PTH (30 min, PTH30), 30 mg/kg bw YKL-05-099 (1 h), 40 mg/kg bw SK-124 (1 h), 10 mg/kg bw 1,25(OH)_2_D_3_ (1 h), or 50 mg/kg bw FGF23 for 1 h. Additional details as for Figure 2. Genomic region displayed is chr2: 170,278,379 to 170,324,235. bw, body weight; ChIP-Seq, chromatin immunoprecipitation sequencing; PTH, parathyroid hormone.

These genomic consequences that we have observed thus far have not included an actual connection to transcriptional output. Fortunately, we can measure the instant impact on transcription, long before enough mRNA accumulates to be measured by quantitative RT–PCR (qRT–PCR). Enrichment of basal machinery chromatin-modifying proteins, such as BRD4, RNA pol II, and histone markers like histone H3 lysine 36 trimethylation or histone H4 lysine 20 monomethylation as well as techniques such as GRO-Seq can provide an idea of the increases or decreases in transcription that is occurring in near real time (33, 34). We decided to monitor BRD4, RNA pol II, and H3K36me3 during basal homeostasis of the mouse (Veh) and our treatments with PTH, YKL-05-099, SK-124, 1,25(OH)_2_D_3_, and FGF23. In Figure 10*A*, we found that the BRD4 occupancy around the *Cyp27b1* locus increased with PTH, YKL-05-099, and SK-124, the activators of *Cyp27b1* activity. The levels of BRD4 are decreased by the suppressors 1,25(OH)_2_D_3_ and FGF23. RNA pol II shows a similar profile as BRD4 for PTH, YKL-05-099, and SK-124. Interestingly, there is a small increase of RNA pol II recruitment with 1,25(OH)_2_D_3_ near the M1 enhancer and within the gene body. 1,25(OH)_2_D_3_ also increased CRTC2 as we showed earlier (Fig. 7). We have included another enrichment region of exon 3 in the gene body for both RNA pol II and H3K36me3 enrichment. This region, exon 3, showed an increase for the activators (PTH, YKL-05-099, and SK-124) threefold to fivefold (Fig. 10*C*). The *Cyp24a1* locus showed the inverse for these hormone profiles, whereby PTH, YKL-05-099, and SK-124 decreased BRD4 (Fig. 11*A*), RNA pol II (Fig. 11*B*), and H3K36me3 (Fig. 11*D*). The 1,25(OH)_2_D_3_ and FGF23 strongly increased BRD4 (Fig. 11*A*), RNA pol II (Fig. 11*C*), and H3K36me3 (Fig. 11*D*). These data indicate that each of these hormones or drugs have elicited dramatic changes in genomic occupancy of key transcription-related factors that ultimately mirror changes in mRNA abundance.

## Discussion

The exquisite control of vitamin D metabolism contains a cascade of genomic responses that quickly corrects any fluctuations in Ca and phosphate levels in the body. A slight decrease in Ca sensed by the parathyroids releases PTH to upregulate *Cyp27b1* expression in the kidney, thus increasing the circulating 1,25(OH)_2_D_3_ (12, 13). This 1,25(OH)_2_D_3_ acts through the VDR to change expression of genes involved in Ca and phosphate regulation in the main mineral-regulating tissues of intestine, bone, and kidney (3, 35). We can model this behavior *in vivo* through injection of PTH, and we find that this activating signal is complex and quite rapid on the genome. Looking first at the mode of “gene activation,” we found that PTH activates the phosphorylation and accumulated recruitment of phosphorylated CREB to the genome in as little as 15 min after intraperitoneal injection of PTH and pCREB recruitment appears to peak at 30 min (Fig. 2). These activities are accompanied by the recruitment of coactivators, such as CBP and CRTC2, who together are believed to form a complex to enhance the transcription of CREB target genes (24, 25); CBP containing histone acetyltransferase activity and CRTC2 facilitating complex formation and stabilization on chromatin. The downstream effects of this are manifested by increasing histone acetylation, increasing recruitment of the basal transcriptional machinery (RNA pol II), and increasing mRNA transcripts. To investigate this mechanism and interaction of coactivators, we turned to the SIK inhibitors that are known to activate in a similar, yet muted, fashion to PTH (22, 26). By using these inhibitors, we attempted to isolate part of the signaling network activated by PTH in proximal tubule cells. We found that SIK inhibition allowed for the accumulation of CRTC2, in particular, to the genome around *Cyp27b1* and *Cyp24a1*. However, the activation profile for the YKL-05-099 compound appeared to be quite similar to that of PTH. In fact, we found that YKL-05-099 increased the serum levels of PTH, which could prove to be a troublesome complication for isolating only the SIK inhibition contributions to activation without PTH interference. Fortunately, the structurally unrelated SK-124 agent did not have the same effects on serum PTH as the YKL-05-099 compound (26). The results from SK-124 injections clearly indicate that CRTC2 is recruited to the genome, and this recruitment was not accompanied by CBP as it was after PTH activation. This is probably most striking near the CP where CBP is highly upregulated by both PTH and YKL-05-099, where SK-124 results in a rapid clearance of CBP from this region. In fact, of any factor we have analyzed by ChIP or ChIP-Seq in the kidney, this is the first such example (CBP) of any meaningful recruitment near the CP, and we are actively investigating this region. While SK-124 did not increase pCREB, pCREB was also not decreased at our enhancers indicating that sufficient pCREB is likely available to activate transcription. These methods, ChIP-Seq in particular, have their limitations on what can be concluded. It is clear that the genomic occupancy of pCREB, CBP, and CRTC2 is all independently increased after PTH induction, and this appears to be a coordinated and cooperative response to form a complex for transcriptional activation. However, that interaction is only assumed and not guaranteed by the ChIP-Seq methodology. We do know from the literature that this interaction is likely, and therefore, our assumption pushes this correlation toward causality (24, 25). The story is more complicated when attempting to assign a causal role for CRTC2 in transcription. While the ChIP-Seq data are suggestive of this causal link, loss-of-function data are needed to demonstrate a functional role of CRTC2 in PTH- and SIK inhibitor–induced *Cyp27b1* induction. These studies are ongoing and the subject of an upcoming article (under review with the *Journal of Clinical Investigation*) with our collaborators and coauthors Drs Wein and Mannstadt.

While the data for activation of *Cyp27b1* appear to be quite clear, the role of this protein complex in PTH action for the suppression of *Cyp24a1* is far more complicated. There, pCREB is largely unchanged, as is the involvement of CRTC2 (Fig. 3). CBP and the resulting histone acetylation are greatly downregulated, which work to dampen the expression of *Cyp24a1*, so the net result is an increase of 1,25(OH)_2_D_3_ in the serum. YKL-05-099 follows with the PTH pattern for limited involvement of pCREB, CRTC2, and CBP removal. However, SK-124 curiously increases CRTC2 recruitment to the downstream (-21 to -42) region, yet the net result is still a downregulation of *Cyp24a1* likely driven by the removal of CBP. This observation questions the role of CRTC2 as a “coactivator” and how it might be assisting gene transcription in this suppression. It is possible that its recruitment is an artifact of nuclear localization caused by SIK inhibition and CRTC2 is accumulating on the existing CREB bound to these enhancers because of the increased CRTC2 nuclear content. It is also possible that other coactivators like SRC1 and SRC3 are assisting in this activity as both are bound to these enhancers as well. Further examination of this suppression mechanism will need to be completed to fully understand these interactions with more examples from the genome-wide dataset. These genome-wide datasets will be the focus of a forthcoming article that focuses on changes in these CREB module factors across the genome in response to PTH treatment.

We refer to these CREB-bound enhancers in the *Cyp27b1* gene locus as “CREB modules” for activity because of the dominant nature of the PTH activation signaling. While these modules likely have a hierarchy for their activities (with the M1 being the dominant enhancer (10, 11)), it is clear they are all involved in the binding of pCREB and its coactivators. The best illustration of this activity was in the cotreatment of PTH or YKL-05-099 with the suppressive FGF23 or 1,25(OH)_2_D_3_ treatments (Fig. S1). The pCREB, CBP, and CRTC2 activities in the M1 and M21(a–c) enhancers favored the activating signals of PTH or YKL-05-099 for recruitment despite the inclusion of the FGF23 or 1,25(OH)_2_D_3_. CBP at the CP, however, was only increased for PTH and YKL-05-099 alone. The synergistic effect in recruitment of CRTC2 in PTH and YKL-05-099 with FGF23 cotreatment is another curious observation. We were concerned with the genomic occupancy on a short time scale in these studies; therefore, we did not examine the gene expression since the maximal times varied between the four compounds (gene expression maximum was 1 h for PTH, 3 h for YKL-05-099, 3 h for FGF23, and 6 h for 1,25(OH)_2_D_3_). This synergistic activity warrants further investigation by future elongated time courses and doses followed by an examination of gene expression.

*Cyp24a1* activation is dominated, as expected, by 1,25(OH)_2_D_3_ signaling as *Cyp24a1* is upregulated in all known cells and tissues that harbor the VDR and are responsive to 1,25(OH)_2_D_3_ (36, 37, 38). It is the benchmark vitamin D-mediated gene that has several enhancers, which are vital to its activity. The PP region has long been known to be associated with gene activation (16), and we have previously published that the interaction of the -35 and -37 kb enhancers also contributes to the VDR-mediated activation of *Cyp24a1* in most tissues (15, 17). The enhancers located at −21 to −32 kb and -39, -42 kb downstream of the transcriptional start site were found to be active in the kidney and do not appear in most other cell types or tissues (15). Here, we examined RXR for the first time in the adult mouse kidney and found a strong ligand-mediated association with the VDR as we had found in previous studies in cell lines (17, 39, 40, 41). This strong activation was met with increased coactivator activities like an increase of CBP (Fig. 8), SRC1, and SRC3 (Fig. 9). Interestingly, the suppression of *Cyp27b1* (Fig. 7) by 1,25(OH)_2_D_3_ had increased VDR recruitment (11) but was not accompanied by an increase of RXR. This is in striking contrast to the active VDR–RXR heterodimer recruitments we see across the genome of many cell types (39, 40, 41, 42, 43, 44, 45). It is important to note that RXR is still present in the homeostatic basal state of the mouse kidney, so while RXR is not increased, it is however, not decreased either. RXR could still be utilized for the VDR–RXR heterodimer in the mechanism of suppression. Suppression by VDR remains to this day an enigma of inconsistent models.

In both the suppressive mechanism of *Cyp27b1* and the activating mechanism of *Cyp24a1*, CRTC2 was increased on the genome in response to 1,25(OH)_2_D_3_. At *Cyp27b1*, not only the increase was greatest at the M1 enhancer (the dominant enhancer for PTH actions (10, 11)) but also increased at the M21. At *Cyp24a1*, CRTC2 recruitment was elevated across the downstream enhancers (-21 to -42 kb) as well as the PP. Here again, like the *Cyp27b1* activator SK-124, and to a lesser extent PTH, we find CRTC2 binding to the genome in both activating and suppressing modes. As SK-124 induces this CRTC2 binding, it is not facilitating the increased recruitment of CBP near the *Cyp27b1* gene. This is a remarkable divergence in SIK inhibition that is not demonstrated with YKL-05-099 likely as YKL-05-099 is also activating the full PTH signaling cascade as YKL-05-099 increases serum PTH concentrations. CRTC2 in the absence of CBP may be utilizing the SRC1 and SRC3 coactivators to help facilitate the rise in histone acetylation activity observed around the *Cyp27b1* locus. SRC1 and CRTC2 were found to have a relationship in hepatic glucose production (46). In these liver cells, CRTC2 is known to be activated by and interact with the glucocorticoid receptor to help regulate genes involved in gluconeogenesis (47). The response of increased CRTC2 occupancy on the genome to 1,25(OH)_2_D_3_ is a novel observation to our knowledge and warrants further investigation. We do observe that genomic regions near the *Crtc2* gene (data not shown) recruit VDR after 1,25(OH)_2_D_3_ treatment, and we are currently investigating these mechanisms. It is possible CRTC2 aids in the ability of VDR in the kidney to regulate vitamin D metabolism as it does with glucocorticoid receptor to regulate gluconeogenesis in the liver.

**Figure 12:**
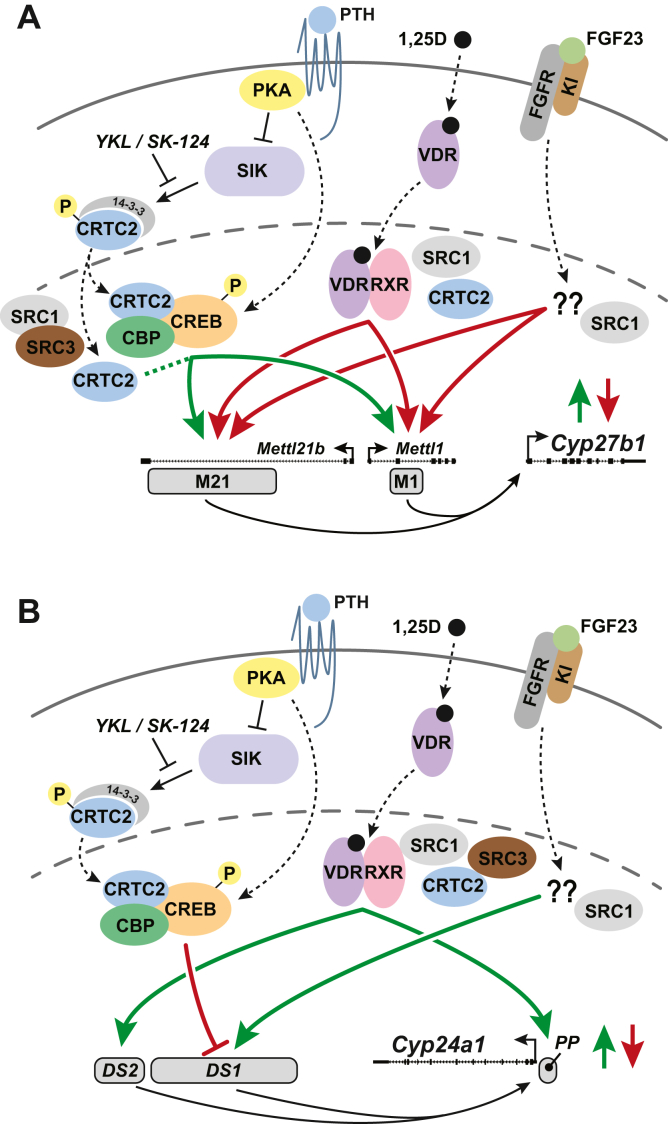
**Summary figure of *Cyp27b1* and *Cyp24a1* regulation in the kidney.** Salt-inducible kinases (SIKs) sequester CRTC2 associated with 14-3-3 proteins in the cytoplasm through phosphorylation of CRTC2. PTH signaling cascade activates *Cyp27b1* expression (*A*) in part by PKA inactivation of SIKs, which allows nuclear translocation of unphosphorylated CRTC2 to complex with CBP and phosphorylated CREB to induce *Cyp27b1* expression (*green up arrow*) through the M1 and M21(a–c) enhancers. SIK inhibition by SK-124 allows translocation of CRTC2 without apparent activation of the PKA pathway or increases of pCREB and CBP to activate *Cyp27b1*. 1,25(OH)_2_D_3_ acts through the VDR and RXR binding at the M1 and M21(a–c) enhancers to suppress *Cyp27b1* (*red down arrow*). SRC1 and SRC3 are involved in PTH/SIK activation, whereas SRC1 is involved in 1,25(OH)_2_D_3_ and FGF23 suppression of *Cyp27b1*. CRTC2 is also increased on the genome by 1,25(OH)_2_D_3_ through an unknown mechanism. FGF23 signaling through the FGFR/Klotho (Kl) membrane receptor leads to suppression of *Cyp27b1* through an unknown transcription factor (??) or signaling cascade. For *Cyp24a1* expression, 1,25(OH)_2_D_3_ and FGF23 signaling uses SRC1, SRC3, and CRTC2 signaling for activation. PTH/SIK signaling suppresses *Cyp24a1* expression (*B*). Enhancers −21 kb to −32 kb, DS1. enhancers −35 kb to −37 kb, DS2. CBP, CREB-binding protein; CRTC2, CREB-regulated transcription coactivator; DS, DownStream region; 1,25(OH)_2_D_3_, 1,25-dihydroxyvitamin D_3_; pCREB, phosphorylated (p-133) CREB; PTH, parathyroid hormone; RXR, retinoid X receptor; VDR, vitamin D receptor.

Overall, these studies have highlighted the very rapid nature of PTH activation of *Cyp27b1* and *Cyp24a1* suppression in the maintenance of vitamin D metabolism, which in turn modulates the delicate serum Ca and phosphate balance. We have summarized these activities in Figure 12, building on the basic models presented in Figure 1. We have demonstrated that these rapid actions involve multiple coactivators in an orchestrated pattern that results in strong activation of *Cyp27b1*. Through these studies, we have created a linkage of exterior cell signaling (Ca and phosphate), through intracellular signaling (signal transduction invoked by PTH through PKA and PKC pathways), to hormonal balance (PTH, FGF23, and 1,25(OH)_2_D_3_), to genomic responses on the chromatin, and finally transcriptional output through recruitment and activation of RNA pol II. Taken together, we have defined an interacting pattern of TFs, coactivators, and histone markers involved with dominant CREB modules that control the reciprocal gene regulation vital to the maintenance of vitamin D metabolism.

## Experimental procedures

### Reagents

The following reagents were used for *in vivo* injections: 1α,25(OH)_2_D_3_ was obtained from SAFC Global, PTH (1–84 human) was obtained from Bachem (H-1370.0100), mouse FGF23 from R&D Systems (2629-FG-025), YKL-05-099 (27), and SK-124 (26). Diluent for the SK-124 was 15% HPBCD (hydroxypropyl β-cyclodextrin; Sigma, catalog no. H107) in sterile water. Antibodies used for ChIP-Seq analysis of VDR (C-20; catalog no.: sc-1008, lot no.: H1216) and RXR (N-197; catalog no.: sc-774, lot no.: D0815) purchased from Santa Cruz Biotechnology, Inc. H3K27ac (catalog no.: ab4729, lot no: GR3374555-1) and H3K36me3 (catalog no.: ab9050, lot no.: GR273247-1) purchased from Abcam. pCREB (catalog no.: 06-519, lot no.: 3460466) and H3K9ac (catalog no.: 06-942, lot no.: 3574473) purchased from EMD Millipore. CRTC2 (catalog no.: A300-737A, lot no.: 1) and BRD4 (catalog no.: A700-004, lot no.: 3) purchased from Bethyl Laboratories. CBP (D6C5; catalog no.: 7389; lot no.: 7), SRC1 (128E7; catalog no.: 2191S; lot no.: 2), and SRC3 (5E11; catalog no.: 2126S; lot no.: 4) purchased from Cell Signaling Technology. RNA pol II (8WG16) purchased from Covance. Traditional genotyping PCR was completed with GoTaq (Promega), and all real-time qPCR was completed with the StepOnePlus using TaqMan for gene expression assays (Applied Biosystems). Primers were obtained from IDT.

### Gene expression

Dissected tissues were frozen immediately in liquid nitrogen and stored at −80 °C. Frozen tissues were homogenized in Trizol Reagent (Life Technologies), and RNA was isolated as per the manufacturer's instructions. About 1 μg of isolated total RNA was DNase treated, reverse transcribed using the High Capacity cDNA Kit (Applied Biosystems), and then diluted to 100 μl with RNase/DNase-free water. qPCR was performed using primers specific to a select set of differentially expressed genes by TaqMan analyses. TaqMan Gene Expression probes (Applied Biosystems) used for RT–PCR were Cyp27b1 (catalog no.: 4351370; Mm01165918_g1∗) and Cyp24a1 (catalog no.: 4351370; Mm00487244_m1).

### ChIP-Seq

ChIP was performed using antibodies listed in reagents. ChIP was performed as described previously with several modifications (48, 49). The isolated DNA (or input DNA acquired prior to precipitation) was then validated by real-time qRT–PCR and further prepared for ChIP-Seq analysis. ChIP-Seq libraries were prepared as previously described (49, 50) with the following exceptions: ChIP-Seq libraries were prepared using the NEBNext Ultra II DNA kit (NEB; catalog no.: E7645S) with the NEBNext Multiplex Oligos for Illumina (NEB; catalog no.: E6440S) according to the manufacturer’s protocols. Libraries were submitted to the University of Wisconsin—Madison Biotechnology Center’s DNA Sequencing Facility (Research Resource Identifier: SCR_017759). Libraries were sequenced on a NovaSeq 6000. Paired-end 250 bp sequencing with a target of 20+ million reads was performed. Data were processed from NovaSeq 6000 with bcl2fastq. For ChIP-Seq FCs (indicated in each figure), ChIP-Seq tag density was evaluated at each enhancer region compared with the tag density for the vehicle treatment. These calculations were processed by HOMER, EdgeR, and DESeq2 (51, 52, 53), and the full table of FCs and raw read density values are included in Table S1.

### Animal studies

C57BL/6 mice aged 8 to 9 weeks (The Jackson Laboratory) were housed in high-density ventilated caging in the Animal Research Facility of University of Wisconsin-Madison under 12 h light/dark cycles at 72 °F and 45% humidity. All mice used in this study were maintained on a standard rodent chow diet (5008; Lab Diet). All experiments and tissue collections were performed in the procedure rooms in the Research Animal Facility of University of Wisconsin-Madison. All animal studies were reviewed and approved by the Research Animal Care and Use Committee of University of Wisconsin-Madison under protocol A005478. Animals were subjected to intraperitoneal injection of 10 mg/kg body weight (bw) 1,25(OH)_2_D_3_ (in propylene glycol), 230 mg/kg bw PTH (1–84) (in PBS), 50 mg/kg bw FGF23 (in PBS + 0.1% bovine serum albumin), 30 mg/kg YKL-05-099 (PBS + 25 mM HCl), 40 mg/kg SK-124 (15% HPBCD), or vehicle (EtOH, PBS, or HPBCD). Animals were sacrificed, and tissues were collected at times indicated in each legend for ChIP and gene expression. Unless otherwise indicated, all experiments were conducted with equal numbers of males and females (n ≥ 6). Data were reported as mixed, as no differences were found between sexes.

### Statistical evaluation

Data were analyzed using GraphPad Prism 9.1.2 software (GraphPad Software, Inc) and in consultation with the University of Wisconsin Statistics Department. All values are reported as the mean ± SEM, and differences between group means were evaluated using One-way ANOVA, two-way ANOVA, or Student’s *t* test as indicated in the figure legends.

## Data availability

All ChIP-Seq data have been deposited in the Gene Expression Omnibus (GSE133025 and GSE206777).

## Supporting information

This article contains supporting information (Supplemental Table S1 as well as Supplemental Figs. S1 and S2).

## Conflict of interest

M. N. W. has consulted for AstraZeneca, Guidepoint, and Galapagos and receives research support from Radius Health. All other authors declare no conflicts of interest with the contents of this article.

## Figures and Tables

**Supplemetal Figure S1:**
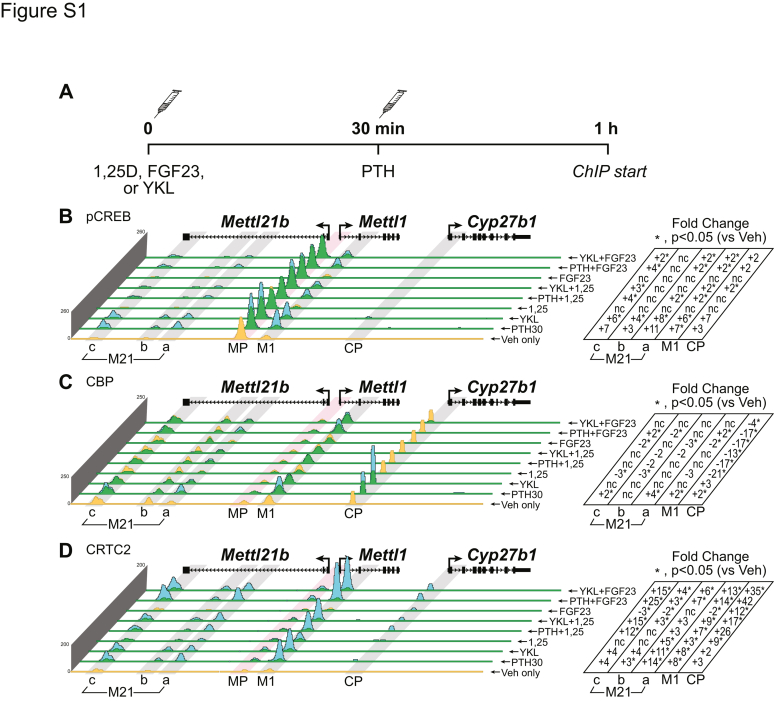


**Supplemetal Figure S2:**